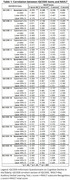# Correlation between the Short Version of Informant Questionnaire on Cognitive Decline in the Elderly and Rey Auditory Verbal Learning Test in an Outpatient Memory Clinic

**DOI:** 10.1002/alz70857_107345

**Published:** 2025-12-24

**Authors:** Julia Cardoso Costa, João Vitor da Silva Viana, Leonardo Ryuiti Kimoto, Giovanna Correia Pereira Moro, Thiago Vilella Daher Bonifacio de Menezes Oliveira, Aline Siqueira de Souza, Joice Coutinho de Alvarenga, Gabriela Tomé Oliveira Engelmann, Bruna Fulgêncio Dias, Marcilene Anacleto Melgaço, Antônio Augusto Rocha Melo Vieira, Marco Aurélio Romano‐Silva, Jonas Jardim de Paula, Maria Aparecida Camargos Bicalho, Bernardo de Mattos Viana

**Affiliations:** ^1^ Cog‐Aging Research Group, Belo Horizonte, Minas Gerais, Brazil; ^2^ Undergraduate Medicine, Faculty of Medicine, Universidade Federal de Minas Gerais (UFMG), Belo Horizonte, Minas Gerais, Brazil; ^3^ Cog‐Aging Research Group, Universidade Federal de Minas Gerais (UFMG), Belo Horizonte, Minas Gerais, Brazil; ^4^ Sciences Applied to Adult Health Postgraduate Program, School of Medicine, Universidade Federal de Minas Gerais (UFMG), Belo Horizonte, Minas Gerais, Brazil; ^5^ Older Adult's Psychiatry and Psychology Extension Program (PROEPSI), School of Medicine, Universidade Federal de Minas Gerais (UFMG), Belo Horizonte, Minas Gerais, Brazil; ^6^ Molecular Medicine Postgraduate Program, School of Medicine, Universidade Federal de Minas Gerais (UFMG), Belo Horizonte, Minas Gerais, Brazil; ^7^ Molecular Medicine Program, School of Medicine, Federal University of Minas Gerais, Belo Horizonte, Minas Gerais, Brazil; ^8^ Neurotec R National Institute of Science and Technology (INCT‐Neurotec R), Faculty of Medicine, Universidade Federal de Minas Gerais (UFMG), Belo Horizonte, Minas Gerais, Brazil; ^9^ Department of Psychiatry, School of Medicine, Federal University of Minas Gerais, Belo Horizonte, Minas Gerais, Brazil; ^10^ Geriatrics and Gerontology Center Clinical Hospital of University of Minas Gerais, Belo Horizonte, Minas Gerais, Brazil; ^11^ Department of Internal Medicine, School of Medicine, Federal University of Minas gerais, Belo Horizonte, Minas Gerais, Brazil; ^12^ Older Adult's Psychiatry and Psychology Extension Program Federal University of Minas Gerais, Belo Horizonte, Minas Gerais, Brazil

## Abstract

**Background:**

The short version of the Informant Questionnaire on Cognitive Decline in the Elderly (IQCODE‐SV) is a widely used questionnaire for screening Dementia. On the other hand, The Rey Auditory Verbal Learning Test (RAVLT) is considered the gold standard to assess episodic verbal memory, a key feature for the diagnosis of typical Alzheimer's Disease Dementia (ADD). This abstract objectives to correlate IQCODE items to RAVLT subscores A6, A7, Recognition, and total score.

**Method:**

This is a cross‐sectional study, using data from the Cog‐Aging cohort study from 2023 and 2024. Participants that underwent clinical geriatric IQCODE‐SV‐BR and neuropsychological evaluations with RAVLT were included. RAVLT standardized “z‐scores” for age and schooling years were correlated to the IQCODE‐SV‐BR scores using a Spearman's correlation test. *p*‐value of <0.05 was used for statistical significance.

**Result:**

Eighty‐seven participants, 70.1% women, with a mean age of 78.08 years (SD 6.391) and median of 4 years of education (IQR 8) were included. The items 1, 6 and 14 of IQCODE‐SV‐BR were the only ones that had significant correlation to all RAVLT scores. Both items 1 and 14 had a mean rho of ‐.33, Item 1 (Min ‐0.28 ‐ Max ‐.387), item 14 (Min ‐0.24 ‐ Max ‐.42), and item 6 a mean rho of ‐.26 (Min ‐.24 ‐ Max ‐.31). Only item 14 had a moderate inverse correlation, which was to A7 score. All other inverse correlations were significant, but only weak. (Table 1)

**Conclusion:**

The IQCODE‐SV‐BR items 1 (“Remembering things about family and friends e.g. occupations, birthdays, addresses”), 6 (“Remembering where things are usually kept”) and 14 (“Handling financial matters e.g. the pension, dealing with the bank”) had a significant negative, but weak correlation with RAVLT scores. This exploratory study identified these three items as the most correlated measures with an objective assessment of episodic memory. Further research with larger samples may clarify whether an even shorter version of the IQCODE‐SV‐BR outperforms the full version in assessing Amnestic Mild Cognitive Impairment or typical ADD.